# Understanding LOT-CRT: Current Insights, Limitations, and Our Center’s Experience

**DOI:** 10.3390/jcm14093025

**Published:** 2025-04-27

**Authors:** Georgios Leventopoulos, Kassiani-Maria Nastouli, Maria Bozika, Eleni Papastavrou, Anastasios Apostolos, Rafail Koros, Angelos Perperis, Ioanna Koniari, Niki Vlassopoulou, Panagiotis Chronopoulos, Christoforos K. Travlos, Athanasios Moulias, Periklis Davlouros

**Affiliations:** 1 Department of Medicine, Division of Cardiology, University Hospital of Patras, 265 04 Patras, Greece; kassienmarie@gmail.com (K.-M.N.); mariabozika29@gmail.com (M.B.); elenipao@gmail.com (E.P.); korosraf@hotmail.com (R.K.); angperperis@upatras.gr (A.P.); iokoniari@yahoo.gr (I.K.); nvlasopoulou@upatras.gr (N.V.); takaroschr@gmail.com (P.C.); dramoulias@live.com (A.M.); pdav@upatras.gr (P.D.); 2 First Department of Cardiology, Hippocration General Hospital, National and Kapodistrian University of Athens, 157 72 Athens, Greece; anastasisapostolos@gmail.com; 3 Liverpool Centre for Cardiovascular Science, Liverpool L14 3PE, UK; 4 Research Institute, McGill University Health Center, Montreal, QC H4A 3J1, Canada; christoforos.travlos@mail.mcgill.ca

**Keywords:** left bundle branch pacing, cardiac resynchronization therapy, LOT-CRT, heart failure, QRS narrowing, intraventricular conduction delay, electrophysiology

## Abstract

Cardiac resynchronization therapy (CRT) using biventricular (BiV) pacing is the standard treatment for heart failure (HF) patients with reduced left ventricular ejection fraction (LVEF) and electrical dyssynchrony. However, one in three patients remains a non-responder. Left bundle branch area pacing (LBBAP) could represent a more physiological alternative, but its effectiveness is limited in cases of atypical left bundle branch block (LBBB) or intraventricular conduction delay (IVCD). Left Bundle Branch Pacing Optimized cardiac resynchronization therapy (LOT-CRT) integrates LBBAP with coronary sinus (CS) lead pacing to improve electrical synchrony and clinical outcomes. This review evaluates the feasibility, advantages, disadvantages, and clinical outcomes of LOT-CRT. Additionally, we describe our center’s experience and propose an evidence-based implantation algorithm. A review of published studies investigating LOT-CRT was conducted, comparing its effectiveness with BiV-CRT and LBBAP alone using QRS narrowing, LVEF improvement, left ventricular remodeling, New York Heart Association (NYHA) class changes and NT-proBNP levels. It was found that LOT-CRT outperforms BiV-CRT or LBBAP alone in selected populations, at the cost of higher clinical skills, longer procedural times, and specific device setups. Randomized trials are underway to further define its role in clinical practice.

## 1. Introduction

Cardiac resynchronization therapy (CRT) using biventricular (BiV) pacing, which combines right ventricle (RV) pacing and left ventricular (LV) epicardial pacing via a coronary sinus branch, is considered to be the gold standard in patients with heart failure with reduced left ventricular systolic function and wide QRS [1]. Nonetheless, CRT implantation is limited in certain clinical scenarios. These include the presence of scar in the left lateral ventricular myocardium, implying high LV lead pacing output or non-capture [2], suboptimal LV lead implantation in an apical position [3], and phrenic capture by LV lead pacing. Considering all these limitations, it is not a surprise that one in three patients does not experience an improvement in symptoms and LV function and is considered a CRT non-responder (a non-responder is defined as having LV end systolic volume reduction <15%) [4]. Conversely, the incidence of super-responders to CRT has been reported to range from 7.3% to 40% [5]. The definition of ‘super-responder’ varies. It defines a patient who marks improvement in left ventricular ejection fraction (LVEF)—typically a normalization or near-normalization to ≥50%, or an absolute increase of ≥30% from baseline—along with significant reverse remodeling of the left ventricle, including reductions in end-systolic and end-diastolic volumes [5].

Left bundle branch area pacing (LBBAP) has emerged as a novel physiological promising alternative to conventional biventricular pacing (BiV-CRT) with the potential to address and overcome the limitations associated with BiV-CRT in special populations [6]. However, there are many cases where LBBAP implantation is restricted or fails to achieve the desired outcome. These limitations include conduction delay in the distal left bundle branch (LBB) [7] or the presence of atypical left bundle branch block (LBBB) morphology [8]. LBBAP-optimized cardiac resynchronization therapy (LOT–CRT) is a combination treatment that utilizes the benefits of traditional CRT with coronary sinus pacing and the advantages of LBBAP [9]. This approach aims to address and offset the limitations shown both by LBBAP and the conventional BiV-CRT.

In this review, the process of the LOT-CRT implantation procedure, the intracardiac lead and device configuration depending on the indication will be discussed. Outcomes of recent trials investigating the feasibility of LOT-CRT in routine clinical settings, clinical scenarios where the technique is utilized, and the advantages and disadvantages of this novel approach will be presented as well. To conclude, we will share our center’s experience, provide cases where LOT-CRT offers a true benefit over conventional BiV-CRT, and suggest an algorithm outlining our standardized approach to the LOT-CRT procedure.

## 2. CRT Indications

Ιn accordance with the 2021 European Society of Cardiology (ESC) Guidelines on cardiac pacing and cardiac resynchronization therapy, CRT is recommended for patients with symptomatic heart failure classified as New York Heart Association (NYHA) functional Classes II–IV, with reduced LVEF (LVEF ≤ 35%) and QRS duration ≥ 130 ms (Class I, Level A). Given the limited evidence supporting CRT in patients with NYHA Class I—derived primarily from subgroup analyses such as MADIT-CRT—the guidelines generally do not recommend CRT in NYHA Class I patients (Class IIb, Level C) [10].

Additionally, CRT is strongly recommended (Class I, Level A) in patients with heart failure and reduced LVEF (<40%) who have a concomitant indication for ventricular pacing due to high-degree atrioventricular (AV) block, independent of NYHA functional class. This includes patients presenting with atrial fibrillation (AF) [10]. Furthermore, for patients with permanent AF who do not achieve effective biventricular pacing (>90–95%) due to conducted AF, CRT combined with AV junction ablation should be considered (Class IIa, Level B). CRT is recommended for patients with symptomatic AF and severely reduced LVEF (≤35%) undergoing AV junction ablation, irrespective of QRS duration (Class I, Level B) [10]. It should be clarified that CRT becomes a definitive, non-reversible therapeutic pathway once AV node ablation is performed. Prior to this, pharmacological management is typically attempted to reduce the AF burden.

Recent evidence suggests that, in specific patient populations with AF and an indication for CRT, pharmacological therapy alone may be efficient in reducing AF burden. Agents such as Angiotensin Receptor Blocker’s (ARBS), Sodium–Glucose Co-Transporters-2 inhibitors, Angiotensin Receptor Neprilysin Inhibitors (ARNI) and vericiguat have been shown to attenuate oxidative stress, a key contributor to the pathophysiology of AF. Thus, by alleviating myocardial stress, these therapies may reduce AF burden, potentially delaying or even obviating the need for device implantation in certain populations [11].

According to the 2022 ESC Guidelines on ventricular arrhythmias and sudden cardiac death, when an implantable cardioverter defibrillator (ICD) is indicated, it is recommended to evaluate whether the patient could benefit from CRT-D (defibrillator) (Class I, Level C) [12]. Additionally, routine early CRT or ICD implantation is not recommended within the first 40 days following an acute myocardial infarction (Class III, Level A). Instead, reassessment of left ventricular function to evaluate for indications for primary prevention ICD or CRT implantation should occur at least 40 days post-myocardial infarction (Class I, Level C), allowing differentiation between transient myocardial stunning and permanent remodeling [12].

## 3. Conduction System Pacing (CSP) Indications

According to the guidelines provided by the ESC on 2023, the conduction system pacing (CSP, including LBBAP and His Bundle Pacing) is not currently indicated as a primary intervention. Currently, CSP and particularly His Bundle Pacing should be considered an option when coronary sinus lead implantation is unsuccessful (Class IIa, Level of evidence B). In patients undergoing His Bundle Pacing (HBP) implantation, an additional right ventricular backup lead should be considered in cases such as pacemaker dependency, infranodal block, high pacing threshold, high-grade AV block, or planned atrioventricular junction ablation. (Class IIa, Level of Evidence C). Additionally, HBP with a ventricular backup lead may be beneficial in patients in need of ‘pace and ablate’ strategy for rapidly conducted supraventricular arrhythmias, especially in the case of a narrow intrinsic QRS. (Class IIb, Level of Evidence C) It can also be considered an alternative to right ventricular pacing in patients with atrioventricular block LVEF greater than 40%, especially those expected to have more than 20% ventricular pacing (Class IIb, Level of Evidence C).

Data regarding LBBAP utilization in clinical practice were originally presented in the most recent guidelines by the Heart Rhythm Society (HRS), the Latin-American HRS (LAHRS) and the Asia Pacific HRS (APHRS). Chung et al. composed a clinical practice guide for the consideration of CSP in a clinical setting. According to these guidelines, CSP is strongly indicated as an alternative when CRT fails to attain the desired outcome [13]. Particularly, it can be beneficial in cases where CRT procedure cannot be achieved (IIa). It might be reasonable for patients with non-LBBB and NYHA classification of III or IV (IIb), in LBBB patients and NYHA II or IV (IIb) and in patients with pacing-induced cardiomyopathy with a high burden of right ventricular pacing who are classified as NYHA II or III it might be a reasonable choice [7,14,15,16,17]. It must be emphasized that additional multicenter trials are necessary to strengthen the evidence base of LBBAP technique.

To add to this, the European Heart Rhythm Association (EHRA), in 2025, presented a consensus regarding CSP pacing [18]. CSP, which includes HBP and LBBAP, is increasingly recognized as a physiological alternative to right ventricular pacing (RVP) and BiV-CRT, particularly in patients with AV block and preserved or mildly reduced LVEF. LBBAP is preferred over HBP in the presence of infra-Hisian block, post-transcatheter or surgical aortic valve replacement, or when lower pacing thresholds and stable sensing are desired. In patients with heart failure and CRT indications, LOT-CRT offers a promising alternative to conventional BiV-CRT. This is especially indicated in specific patients such as those with typical LBBB, suboptimal response to traditional CRT, or failed coronary sinus (CS) lead implantation. LOT-CRT allows for fusion pacing and may enhance electrical resynchronization by targeting both septal and lateral ventricular activation. In the case of patients undergoing atrioventricular node ablation (“pace-and-ablate” strategy), CSP is also recommended to preserve synchrony and avoid pacing-induced dysfunction. Overall, these updated recommendations highlight the expanding role of CSP, and particularly LOT-CRT, in tailoring CRT delivery for improved physiological outcomes.

## 4. LBBB Definitions

Given the importance of defining the LBBB morphology to determine the eligible CRT recipients, it is essential to clarify the current diagnostic criteria. As we will discuss later, the type of LBBB (typical vs. atypical) may reflect different sites of block, which, in turn, have an impact on electrical resynchronization when an LBBAP strategy is adopted. Therefore, it is useful at this point to describe how typical LBBB is characterized by the most recent guidelines.

To LBBB morphology, certain criteria must be met. According to the American College of Cardiology/American Heart Association/Heart Rhythm Society (ACC/AHA/HRS) guidelines, the QRS duration must be >120 ms; there should be notched or slurred R waves in leads I, aVL, V5, V6, or absent Q waves in leads I, aVL, V5, V6; the ST and T waves should be in the opposite direction of the QRS; the R peak time > 60 ms in V5 and V6 and normal in V1, V2, V3. Moreover, there may be ST segment depression in leads with negative QRS and/or negative T waves in the same leads. LBBB may change the mean QRS axis and in leads with positive QRS, and a positive T wave may be considered normal, reflecting positive concordance [19].

In addition to these, ESC defines LBBB morphology as a QRS duration >120 ms, notching or slurring of QRS, with an R peak > 60 ms in V5 and V6, a variable QRS axis, and ST opposition to the QRS—especially when QRS duration exceeds 140 ms. A QS or rS pattern in V1 with a small r wave in V1 is also noted [20].

According to a study conducted by Strauss et al., the criteria for the characterization of LBBB morphology include sex-specific QRS durations (>140 ms for men and >130 ms for women), the presence of mid-QRS notching or slurring in >2 contiguous leads, and a negative terminal deflection in lead V1 manifesting as a QS or rS morphology [21]. In Table 1 the definitions of LBBB are presented according to the Strauss criteria, the ESC guidelines of 2021 and the 2018 ACC/AHA/HRS Bradycardia Clinical Practice Guidelines Executive Summary on the evaluation and management of patients.

## 5. Technical Challenges on LBBAP

LOT-CRT is a novel approach in biventricular pacing, introduced by Vijayaraman [9]. In essence, LOT-CRT resembles a conventional CRT procedure, with the key distinction of replacing the RV pacing lead by a LBBAP lead, which enables direct stimulation of the cardiac conduction system.

**Figure 1:**
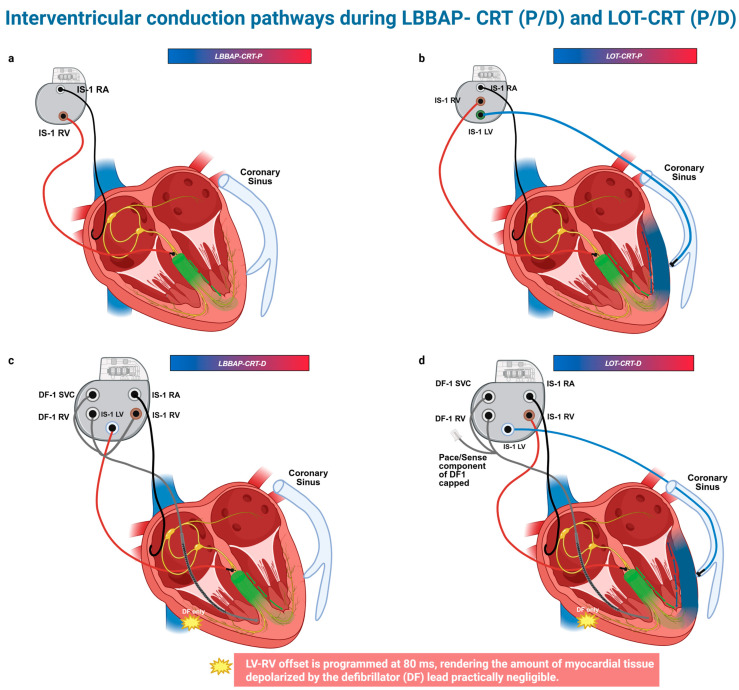
Devices’ configurations and interventricular conduction pathways for LBBAP-CRT (P/D) and LOT-CRT(P/D). (**a**) LBBAP-CRT-P, (**b**) LOT-CRT-P, (**c**) LBBAP-CRT-D, (**d**) LOT-CRT-D. This schematic illustration depicts the conduction pathways involved in LBBAP- CRT and LOT-CRT. The green gradient line represents the electrical impulse generated by the LBBAP lead. LV depolarization originates solely from the LBBAP lead. In contrast, in LOT-CRT, the blue line illustrates the electrical impulse originating from the LV epicardial lead. In a LOT-CRT device, the LV is depolarized in a fusion mode, by an impulse propagating from the LBBAP lead (green gradient line) and the LV epicardial lead (blue gradient line). Figure created in BioRender. Bozika, M. (2025) https://BioRender.com/25t1kd2 (accessed on 12 April 2025).

At this point, to ensure clarity and prevent potential confusion, it is important to precisely define the resynchronization techniques referenced throughout this manuscript. LBBAP-CRT-P refers to the left bundle branch pacing with a pacemaker device (Figure 1a), involving a single lead positioned for conduction system pacing. LOT-CRT-P denotes a left bundle branch optimized CRT approach that combines LBBAP with an additional pacing lead placed in the CS (Figure 1b) to enhance ventricular synchrony, utilizing a dual-site pacing configuration. LBBAP-CRT-D describes LBBAP in conjunction with a defibrillating lead positioned in the right ventricle, implemented as part of a cardiac resynchronization therapy defibrillator (CRT-D) system (Figure 1c). Finally, LOT-CRT-D represents the combination of LBBAP, CS pacing, and a right ventricular defibrillating lead, forming a comprehensive CRT-D approach (Figure 1d). Additionally, Figure 1 depicts interventricular conduction pathways in the above modalities. These terms are used consistently throughout this manuscript to represent their respective techniques.

LBBAP is implemented by inserting a lumenless or stylet-driven lead combined with a dedicated sheath, deep into the interventricular septum, 1–2 cm from the distal His Bundle [22]. A backup lead should be inserted in the RV in LBBB patients to prevent complete heart block in case of transient injury to the right bundle. The optimal position for the LBBAP lead is determined by the presence of electrophysiologic markers, which are considered direct evidence of LBBAP capture [23].

As described by Wu et al., this is a time-consuming and complex process which necessitates the placement of (a) another lead at the His position and (b) a multielectrode catheter in the LV septum area. Direct evidence of LBB capture is established when left ventricular conduction system potentials (distal to the site of pacing) are distinctly recorded and precede the ventricular electrograms on the multielectrode catheter. Moreover, in the case of LBBB and His correction pacing, LBB capture is evident when the stimulus to LV activation time (S-LVAT) during LBBAP is shorter than S-LVAT during HBP [23].

It is evident that such a process is impractical for routine clinical application of LBBAP. To address this limitation, the EHRA consensus statement [22] has proposed several criteria with defined cutoff values, some of which act as surrogate markers for LBB capture. Additionally, the recording of Left Bundle potentials—one of the primary criteria for LBBAP capture—is often absent in patients with LBBB, owing to the intrinsic pathophysiology of the conduction disorder. A commonly utilized maneuver to confirm LBB capture involves observing a transition from selective to non-selective LBB capture, or from non-selective to left ventricular septal pacing (LVSP), typically characterized by an abrupt increase in the Stim-LVAT interval [22]. However, this transition was observed in only 26.4% of cases in the MELOS registry [24], likely due to the similarity between conduction and myocardial tissue capture thresholds in most patients. Therefore, the validation of LBB capture in the context of LBBB primarily relies on ECG-paced features and surrogate electrophysiological criteria. These include RBBB morphology in lead V1, accompanied by a short pacing stimulus to LV activation time (Stim-to-LVAT < 80 ms) and a prolonged interpeak interval (R in V6 to R′ in V1 > 44 ms) [22]. These considerations highlight the importance of a comprehensive approach to LBB capture verification, underscoring that no single parameter is sufficient and that confirmation often demands significant electrophysiological expertise.

**Figure 2:**
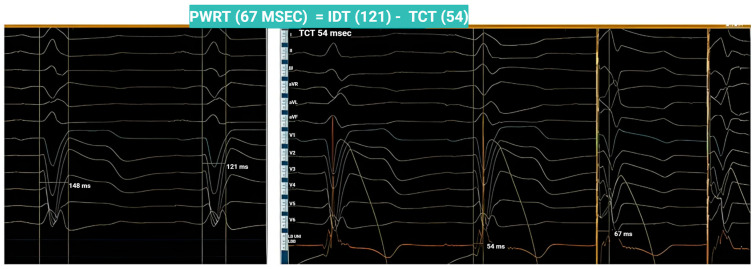
A paradigm of the physiological criteria in a patient with intrinsic QRS 148 ms with LBBB morphology. The intrinsicoid deflection was recorded at 121 ms, corresponding to the left ventricular activation time. Upon advancing the LBBAP lead into the interventricular septum, the transsptal conduction time (TCT) was determined by measuring the interval from QRS onset to the maximum peak deflection recorded by the LBBAP lead (54 msec). Ideally, the paced SLVAT (left ventricular activation time during pacing) should equal the intrinsic activation time minus the TCT, as demonstrated in this case. This finding suggests the elimination of the abnormal right-to-left septal activation delay, supporting successful LBB capture. These observations are consistent with the physiological criteria for LBB capture in LBBB patients, as described by Jastrzębski et al. [25].

Moreover, Jastrzębski et al. have proposed a set of physiological criteria for confirming LBB capture, emphasizing that the principal objective of LBBAP in patients with LBBB is to accurately determine the timing of the electrical stimulus as it propagates through the interventricular septum from right to left. Figure 2 depicts a conceptual paradigm grounded in these physiological criteria [25].

Apart from the challenges related to defining LBB capture, LBBAP is a technically demanding technique with strict criteria for confirming LBB capture [22]. This is crucial if the patient receives a device for resynchronization. The ideal pacing site lies within a narrow subendocardial zone of the left ventricular septum, making precise lead placement crucial.

Within the framework of conduction system pacing, the term LBBAP has been used broadly to include, left bundle branch pacing (LBBP), left fascicular pacing (LFP), and LVSP. While LBBP and LFP differ only slightly—both signifying successful conduction system capture—LVSP represents a distinct entity with inferior electrophysiological and clinical outcomes, as described by Diaz et al. [26]. Despite being grouped under the broader LBBAP category, LVSP should be recognized as a separate and less optimal endpoint. Not clearly distinguishing these may lead to clinical ambiguity. Furthermore, this categorization highlights how procedural challenges may result in acceptance of LVSP as a technically achievable, yet suboptimal solution, still falling within the broader definition of LBBAP.

Failure to achieve this target zone often results in prolonged S-LVAT intervals—reflecting delayed left ventricular activation time and reduced resynchronization outcome. While pacing in this region may yield an RsR’ pattern in lead V1 (as in LBBAP), this finding is not specific for LBBAP, as it can also occur in LVSP. Conversely, the absence of RsR’ pattern does not exclude LBBAP capture. This limitation is addressed in the study by Pujol Lopez et al. [27], who proposed the term “left bundle branch capture plus (LBBC-plus)” criterion.

Other factors can also contribute to suboptimal outcomes. Anatomical difficulties such as scar tissue encountered during septal penetration may hinder lead advancement. In such cases, even if the lead is adequately positioned, the SLVAT may remain prolonged. Scar tissue is characterized by low conduction velocity compared to normal myocardial tissue, which has high conduction velocity, translating to a longer SLVAT and prolonged activation [28].

**Figure 3:**
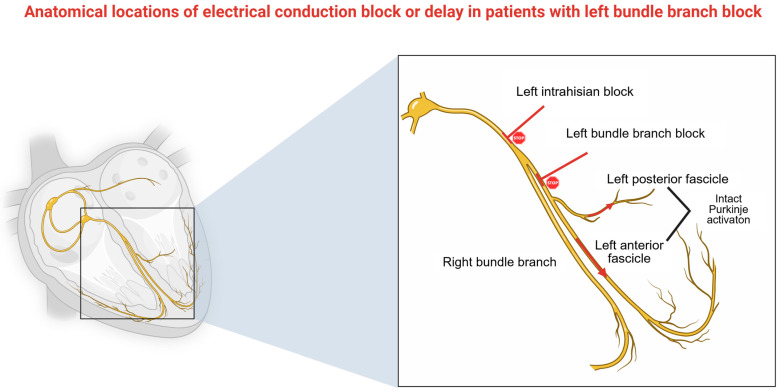
Visual representation of conduction block sites. In case of left Infrahisian block or LBBB, which are present in 46% and 18%, respectively, there is a high likelihood of LBBB correction. On the other hand, in 36% of cases with LBBB, Purkinje activation appears intact but slow. On this occasion, LBBB correction is not possible. Figure created in BioRender. Bozika, M. (2025) https://BioRender.com/kloieup (accessed on 12 April 2025).

The primary objective of LBBAP is to restore physiological ventricular activation by capturing conduction fibers distal to the block, particularly in patients with LBBB. However, this is not always achievable. Upadhyay et al. demonstrated that the effectiveness of His Bundle Pacing, and by extension LBBAP, is closely tied to the block’s anatomical location [29]. LBBAP is most effective when the block is proximal at the His region or the proximal segment of the left bundle (complete conduction block—CCB). However, in 36% of LBBB cases, the block is located distally, or distal Purkinje activation remains intact (IPA), resulting in QRS prolongation, due to the slow interventricular conduction and dilated, myopathic LV. A visual representation of the sites of the block is depicted in Figure 3.

Although ECG criteria—such as the “strict” Strauss criteria—are applied to infer block location, their predictive accuracy is limited. Upadhyay et al. demonstrated that while 91% of patients with CCB and 39% of those with IPA met Strauss criteria, the positive predictive value for QRS correction with His bundle pacing was only 71% [29]. No equivalent study has yet associated this predictive value for LBBAP, though outcomes may be similar or slightly inferior given its more distal pacing site These findings suggest that LBBAP alone may not achieve optimal resynchronization in all cases, particularly in operator-dependent scenarios (i.e., inadequate lead advancement), tissue-related limitations (i.e., myocardial scar), or cases of diffuse conduction disease (i.e., distal conduction block). In such settings, the addition of an LV lead may be necessary to augment therapy.

It becomes evident that multiple factors can influence the outcome of an LBBAP procedure. As previously discussed, defining appropriate LBBAP criteria in patients with LBBB remains challenging. LVSP capture is considered suboptimal in the context of resynchronization therapy. Furthermore, anatomical constraints and the presence of a distally located conduction block may limit the effectiveness of CSP as a standalone approach, thereby necessitating the implementation of an LOT-CRT strategy. Thus, LOT-CRT may represent a more effective alternative than conventional CRT for this subset of patients, as supported by published studies and our initial single-center experience.

## 6. Current Studies and Clinical Significance

In 2021, Vijayaraman et al. [9] reported the case of an 81-year-old patient with heart failure (HF) (LVEF 20%, QRS 210 ms) in whom HBP alone was insufficient to provide adequate correction. An additional LV lead and LBBAP lead were implanted, reducing QRS to 144 ms. At the six-month follow-up, the LVEF had improved to 30%, and the patient’s NYHA status improved from III/IV to II.

Following this, several studies began investigating its feasibility and effectiveness. LOT-CRT demonstrated greater QRS reduction compared to conventional BiV pacing. While preliminary data exist, the current studies lack long-term endpoints, particularly concerning all-cause mortality and heart failure rehospitalizations. Table 2 summarizes the findings on QRS reduction, NYHA class, NT-proBNP, LVEF, echocardiographic parameters, and reported clinical outcomes.

Jastrzębski et al. [30] conducted a prospective, single-arm, multicenter feasibility study enrolling patients who (a) qualified for CRT or (b) CRT non-responders. While the patients received both LOT-CRT-D and LOT-CRT-P, the data provided are not separately presented for both modalities. Regarding mortality, one heart-failure-related death was reported, along with two non-cardiac deaths. Data on heart failure hospitalization were unavailable. No major long-term complications were observed.

Chen et al. [31] conducted a study comparing LOT-CRT with BiV-aCRT (optimized BiV-Pacing) in patients with heart failure with reduced ejection fraction (HFrEF) (LVEF < 35%) and LBBB. LOT-CRT was associated with a greater reduction in QRS duration in comparison to BiV-CRT, corroborating the findings by Jastrzębski et al. In the LOT-CRT group, a super-response group was observed, where LVEF, at 1-year follow-up, had a greater increase. In the current study, the definition of a super-responder was characterized by an absolute increase in LVEF of ≥20% from baseline or attainment of follow-up LVEF ≥ 50%. NYHA status and NT-proBNP levels were also improved, reflecting a reduction in the symptomatic burden ofHF. All the outcomes refer to the LOT-CRT-P modality without including data on LOT-CRT-D. No deaths were reported, and heart failure hospitalizations rates were similar between the two groups (*p* = 0.437) at 1-year follow-up.

In a separate study, Feng et al. [32] introduced an innovative LBBAP approach by integrating an adaptive CRT algorithm in LBBAP therapy, aimed at defibrillation therapy. This system allowed for automatic delivery of LBBAP during periods of normal AV conduction, with a transition to BiV pacing (LOT-CRT) in abnormal AV conduction. They compared the safety and efficacy of LOT-aCRT-D versus BiV-aCRT-D, placing the LBBAP lead in the LV port and the CS lead in the RV pace-sense port. Their findings confirmed the feasibility of LOT-CRT in patients with systolic HF and LBBB, significantly improving QRS duration and echocardiographic parameters. No mortality or major implantation-related complications were reported over a 9-month follow-up. Episodes of ventricular tachycardia (VT)/ventricular fibrillation (VF) were observed but there were no statistical differences between the two groups (*p* = 0.175). Nonetheless, the relatively short follow-up period limits the ability to draw firm conclusions regarding long-term survival benefits.

Parale et al. [33] compared patients with Non-Ischemic Cardiomyopathy (NICM) and LBBB who received LOT-CRT-P versus those who underwent LBBAP-CRT-P, for resynchronization therapy. In addition to conventional clinical outcomes, the study assessed T-peak to T-end (TpTe) and the derived TpTe/QT ratio, both established markers of arrhythmic risk. Among patients with a baseline QRS duration greater than 131 ms under LBBAP-CRT, LOT-CRT achieved a significantly greater reduction in QRS duration (153 ± 19.7 ms vs. 140.2 ± 17.6 ms, *p* = 0.03). However, when the QRS duration was of less than 131 ms, the reduction with LOT-CRT was not statistically significant (*p* = 0.24). These findings suggest that in patients with wider QRS complexes, the underlying conduction disorder may not be confined to a discrete block but may also involve diffuse peripheral conduction abnormalities, necessitating the additional QRS reduction provided by LOT-CRT. In such cases, dyssynchrony cannot be fully corrected by LBBAP alone, making the final pacing outcome suboptimal. No data were reported regarding mortality, heart failure hospitalizations, or arrhythmic events.

Chen et al. (2023) [34] conducted a non-randomized trial comparing echocardiographic and clinical outcomes in patients receiving LOT-CRT with those receiving BiV-CRT, with a greater follow-up period, of 24 months, for resynchronization and defibrillation therapy. The novelty of this study lies in the fact that the patients did not present with LBBB but interventricular conduction delay. This is a subgroup in which LBBAP exclusively is unlikely to restore physiological conduction due to the block being distal to the pacing site. Therefore, LOT CRT was proposed as a more promising option. In the LOT-CRT (including both CRT-P and CRT-D) group, the patients experienced superior improvement in left ventricular function and structural remodeling over a two-year follow-up (LVEF increased significantly). It was associated with a significant reduction in heart failure severity, as well as lower rates of all-cause mortality and heart-failure-related hospitalization compared to BiV-CRT (*p* = 0.035). These outcomes support the hypothesis that LOT-CRT may confer a long-term survival benefit in this patient population.

Ezer et al. [35] examined the application of LOT-CRT-D based on an index called the QLV ratio. The QLV ratio was calculated by the QLV (the time interval from onset of Q to LV sensing) divided by the QRS width at baseline. An optimal LV lead implantation is equally related to a long QLV interval. Two different arms were compared. When the QLV ratio was greater than 70%, BiV-CRT patients were accepted as the final pacing modality, while in the case of a QLV ratio of less than 70%, the LOT-CRT strategy was chosen for selective patients. There were no data regarding mortality and hospitalization rates, and there was no sustained ventricular arrhythmia requiring shock therapy.

Ribes et al. [36] compared LOT-CRT-P with LBBAP in patients suitable for CRT-P implantation, with the objective of identifying predictors of greater QRS narrowing. Their analysis revealed that the predictors for better QRS narrowing were left ventricular end diastolic diameter (LVEDD) > 66 mm and NYHA Class 3 (*p* = 0.019), factors that should be considered indicators for LOT-CRT implantation on clinical settings. No data regarding mortality and heart failure hospitalizations were observed.

Recently, the outcomes of the Conduction System Pacing Optimized Therapy (CSPOT) [37] offered a novel perspective on CRT optimization in patients, with intraventricular conduction disease (IVCD) and LBBB. The patients underwent multiple pacing modalities while being hemodynamically stable. The primary endpoint was to evaluate changes in the left ventricular pressure maximal first derivative (LV dP/dt_max_) and to compare the acute hemodynamic and electrocardiographic effects of different pacing modalities, including LOT-CRT-D, BiV-CRT-D and bipolar LBBAP only, unipolar LBBAP only. QRS shortening was greater in LOT-CRT compared to the other modalities but LV dP/dt_max_ had a similar improvement across groups. Successful LBBAP was achieved in 27 out of 48 patients, while the remaining underwent deep septal pacing (DSP), leading to its achievement in the rest of them. Deep septal pacing indicates that the lead was positioned even less optimally than LVSP, further highlighting the high technical demands of this procedure. While both BiV-CRT and LOT-CRT showed similar improvements in dP/dt_max_, the disparity in QRS narrowing highlights the lack of a direct correlation between electrical and mechanical indices of resynchronization. It is essential to remember that resynchronization is primarily a mechanical process aimed at restoring left ventricular pump function, best reflected by dP/dt_max_ rather than QRS duration alone. Thus, these findings reinforce this principle, indicating that improvements in QRS narrowing do not necessarily translate to equivalent hemodynamic benefits.

Additionally, according to the same study, there is a greater benefit from LOT-CRT in comparison to BiV-CRT in IVCD patients. The benefit in QRS shortening was more substantial in IVCD patients in comparison to the LBBB ones. Given that a considerable number of patients, in routine clinical practice do not fulfill the Strauss criteria for LBBB, LOT-CRT may potentially offer a superior alternative to BiV-CRT in achieving electrical resynchronization. When an LBBAP attempt results in crossover to DSP, LOT-CRT may serve to augment the modest resynchronization effect provided by DSP. In this subgroup analysis, it was further evaluated that LOT-CRT showed a greater hemodynamic advantage in patients with DSP with a baseline QRS ≥ 171 ms, reducing QRS to 20.8 ms more with a 14.5% increase in dP/dt_max,_ outcomes that surpass the LBBAP ones. Anodal capture was associated with a diminished contractile response, while QRS reduction was similar, highlighting that electrical synchrony alone does not guarantee a mechanical benefit. However, this analysis primarily focused on acute hemodynamic improvements rather than long-term electrocardiographic data, limiting insights into sustained ventricular function recovery. Additionally, no data on mortality or heart failure hospitalization were provided.

BATTLE (NCT06061627) is an ongoing, prospective, multicenter, randomized controlled trial designed to assess the LV systolic function 6 months post implantation in two different populations. Patients with HF and intraventricular block will receive LOT-CRT and will be compared to another group that will receive BiV-CRT. The trial is anticipated to be completed in 2027, and it is expected to provide additional insight into the efficacy of LOT-CRT in a clinical setting.

RESCUE (NCT06148571) is a currently ongoing trial investigating the efficacy of LBBAP or LOT as a rescue therapy to BiV-CRT in patients for whom conventional pacing may be ineffective or technically infeasible. The study focuses on individuals with heart failure and reduced ejection fraction, and aims to evaluate procedural success, complication rates and long-term clinical outcome, associated with these alternative pacing modalities.

The cumulative outcomes of these studies highlight that LOT-CRT is associated with superior left ventricular function improvement, especially in LBBB patients. Additionally, QRS narrowing was consistently greater in the LOT-CRT group compared to the BiV-CRT. Despite the observed QRS reduction, evidence remains limited regarding the association of QRS narrowing with long-term mortality benefits, or reduced heart failure hospitalizations.

Currently, only a limited number of studies report follow-up longer than 12 months (usually 3–12 months). Chen et al. [34] provide only one of the few datasets with a 24-month follow-up. Their outcomes showed a substantial improvement in LV remodeling, LVEF increase, QRS narrowing and at the same time a reduction in heart failure hospitalization and all-cause mortality in the LOT-CRT group, in the long term, results that are clinically important and underline the need of randomized trials with prolonged follow-up durations.

Moreover, it becomes essentially important to examine how response in CRT is defined and whether commonly used criteria truly reflect clinical and hemodynamic benefit. The most common definition of responders in resynchronization therapy is based on clinical improvement (NYHA class) and echocardiographic parameters (absolute 5% improvement in LVEF and ≥15% decrease in LV-end-systolic volume. This raises the critical question of whether acute electrical improvements directly correlate with mechanical resynchronization. Zucchelli et al. [38] demonstrated that the ratio between QRS duration during biventricular pacing and right ventricular pacing (RVp) [QRS (BiV-CRT)/QRS (RVp)] was the best predictor of LV functional recovery after CRT (AUC = 0.72; 95% confidence limit 0.57–0.82; *p* < 0.001). The CSPOT study demonstrated that an acute reduction in QRS does not always correlate with dP/dt_max_ improvement, underscoring the multifactorial nature of CRT response. Extrapolating this conclusion to LOT-CRT is a strong assumption but needs confirmation.

CRT response may be affected by several factors, one of them being contrast-induced nephropathy (CIN). CIN may occur after CRT implantation due to the use of iodinated contrast for LV lead positioning. Strisciuglio et al. [39] observed, in a retrospective study, that CIN occurred in 12% of CRT recipients and, although it did not affect the overall response rate, it significantly impaired LVEF recovery. Moreover, it was associated with worse survival among responders, highlighting the importance of nephroprotection, especially in procedures with longer duration.

While the current studies provide accumulative outcomes for LOT-CRT, it is, from a clinical aspect, important to further define the differences in the outcomes between pacing only devices (LOT-CRT-P) and those including a defibrillator (LOT-CRT-D). In the current analysis, the data provided for each modality are sparse. The clinical outcomes are presented collectively between the two groups, while only a limited number among them specifies their analysis. Several studies proceed to determine the outcomes of a defibrillator therapy, although there is a lack of information regarding mortality and arrhythmic risk management in the specific populations [32,35]. The majority of studies that focused their outcomes more on the LOT-CRT-P are focusing more on QRS, NYHA and LVEF without providing more articulate data on mortality and longer follow-up periods [31,33,36]. With that in mind, it becomes evident that further evaluation of each modality is needed with separate outcomes for both resynchronization and defibrillator therapy with LOT-CRT.

## 7. Advantages

Based on the outcomes of all available studies, LOT-CRT appears to surpass conventional BiV-CRT and, in many cases, standalone LBBP. Across the studies, LOT-CRT has consistently achieved a significant reduction in QRS duration, facilitating improved ventricular activation and mitigating dyssynchrony-related deterioration in cardiac function. Additionally, LOT-CRT demonstrated improvements in LVEF and NYHA class, correlating with reduced heart failure hospitalizations and mortality compared to traditional approaches. Unlike BiV-CRT, LOT-CRT is not constrained by the site of conduction block, making it a viable option for addressing peripheral conduction disease and IVCD. However, further randomized trials are necessary to solidify its clinical benefits and define its role in routine practice.

## 8. Disadvantages

On the other hand, it is essential to acknowledge the limitations and potential drawbacks of this promising technique. Firstly, its complexity requires advanced expertise, as the lead configuration within the heart and pulse generator demands a deep understanding of LBBAP. Consequently, it is not suitable for inexperienced operators. The learning curve for LBBAP implantation requires at least 50 cases. However, after completing more than 150 procedures, operators experience a significant reduction in procedure duration, which then stabilizes at a plateau [40].

Additionally, as demonstrated by the aforementioned studies, the procedure time is prolonged, with significantly increased fluoroscopy duration and higher contrast volume usage compared to conventional CRT. Furthermore, a key limitation arises when implanting LOT-CRT-D as the capped RV lead renders the patient ineligible for MRI scanning, restricting future imaging options if needed. In the future, this issue may be addressed with specialized defibrillator leads designed for LBBAP positioning. Cases have been reported in which a conventional ICD lead was successfully placed at the LBBAP site using a specialized curved sheath, as well as instances where operators utilized a standard ICD lead for both pacing and defibrillation from the LBBAP position [41,42]. This approach allows the operator to use a single lead for both pacing and defibrillation. Currently, manufacturers are developing specialized, thin ICD leads designed to integrate both functionalities.

CRT-related complications remain a concern, including LV lead-specific issues such as phrenic nerve stimulation, CS dissection, and LV lead dislodgment, similar to conventional CRT. Additionally, specialized equipment is required, which is often challenging to obtain, particularly for LOT-CRT-D. Addressing current limitations, such as MRI incompatibility due to the capped RV lead, necessitates manufacturer support for improved device options. Currently, reliance on outdated DF-1 leads and corresponding generators persist, as newer alternatives remain unavailable. Furthermore, LOT-CRT entails two procedural steps—LBBAP implantation and LV lead placement—exposing patients to both peri-procedural and post-procedural complications associated with LV lead deployment. Table 3 provides details on fluoroscopy and procedure duration across the studies referenced.

## 9. Our Center’s Experience—Proposed Implantation Algorithm

Conduction system pacing and especially LBBAP have emerged in recent years as promising pacing modalities in CRT and in patients with heart failure that need resynchronization. It is mandatory for randomized trials to be conducted so that these can be implemented in the official guidelines, but data from trials that have been conducted so far are encouraging concerning QRS shortening and the improvement in LVEF [5,17,24,34] The same should be considered for LOT-CRT, for which data in the literature are limited. CSPOT gave some insight into its superiority in a particular population sample, but it needs to be further researched.

Currently, in our center, ECHO-LBBp (NCT06689111), an ongoing, non-randomized, interventional multicenter study, is being conducted. Patients requiring resynchronization, undergo either LBBAP or conventional biventricular pacing. As the study is ongoing, there are yet no clinical outcomes to be presented. Though, preliminary data have shown that, 70% received successfully LBBAP, fulfilling capture criteria, while there was a 30% need for crossover to LOT CRT and BiV CRT. These outcomes are consistent with those presented by Zhu et al. [43], where LBBAP was successful in 72/128 patients (56.25%). In contrast to this study, the researchers accepted LVSP as an outcome, while in our center, it is not, which might justify the small difference in the successful implantation percentage. These preliminary data underline the importance of LOT-CRT, which acts as a connecting technique between LBBP and CRT when none of the aforementioned techniques are successful and can be helpful in their respective clinical scenarios.

**Figure 4:**
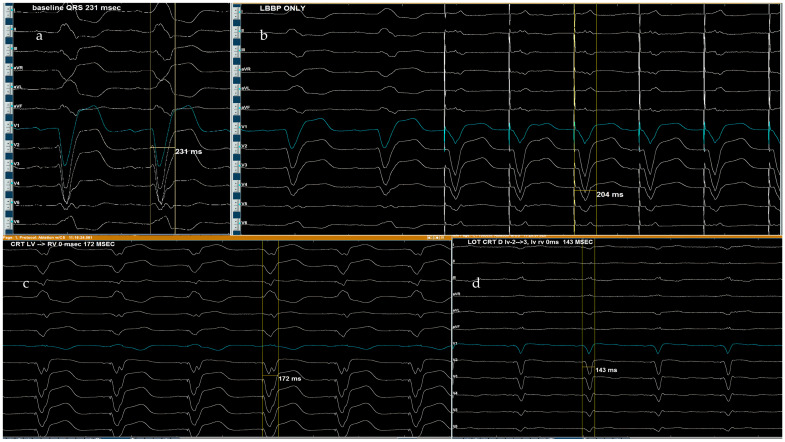
This figure presents serial QRS narrowing, on our center, through different pacing modalities in a patient with a baseline QRS of 231 ms. (**a**) Baseline QRS of 231 ms (native conduction), (**b**) LBBAP only. QRS of 204 ms, (**c**) CRT LV-RV = 0 ms. QRS of 172 ms, (**d**) LOT-CRT. QRS of 143 ms.

In our center, LOT-CRT has been successfully implemented, delivering optimal results. Figure 4 illustrates a case in which a patient with a baseline QRS duration of 231 ms underwent LBBAP, reducing QRS to 204 ms. Subsequent CRT with simultaneous RV-LV pacing further shortened it to 173 ms but remained suboptimal. Finally, LOT-CRT was performed, achieving a further reduction to 143 ms, maximizing electrical resynchronization.

In our center, we prioritize the use of MRI-compatible devices. LOT-CRT-D is not the preferred first-line strategy due to its lack of MRI compatibility.

A possible step-by-step approach and suggested algorithm, based on our experience, is outlined below, depending on the procedure’s objective:

(A) Resynchronization only;

(B) Resynchronization and defibrillation therapy.

(A) Resynchronization therapy:

Initially, a temporary atrial lead is placed in the right ventricle as a backup. The procedure then proceeds with LBBAP implantation. If successful—meaning LBBB correction is achieved with confirmed LBB capture (e.g., S-LVAT < 80 ms, interpeak R difference > 44 ms)—an LV lead is unnecessary, and a dual-chamber pacemaker is implanted as part of a CRT-P-LBBAP strategy. However, if LBB capture and sufficient QRS narrowing are not achieved, crossover to LOT-CRT-P is preferred, adding an epicardial LV lead via the coronary sinus. Finally, the atrial lead is placed in the right atrial appendage.

(B) Resynchronization and defibrillation therapy:

Here, the DF1 lead serves as a backup, eliminating the need for temporary atrial lead placement in the right ventricle. A DF1 lead is generally preferred over a DF4 lead as it allows for crossover to LOT-CRT not only intraoperatively but also in case of late disease progression beyond the LBBAP site. Such progression could negate LBBB correction, necessitating LOT-CRT, which is not feasible with a DF4 lead. Instead, a new DF1 lead would need to be inserted, ideally with extraction of the existing DF4 lead. This configuration, however, is not MRI-compatible.

**Figure 5:**
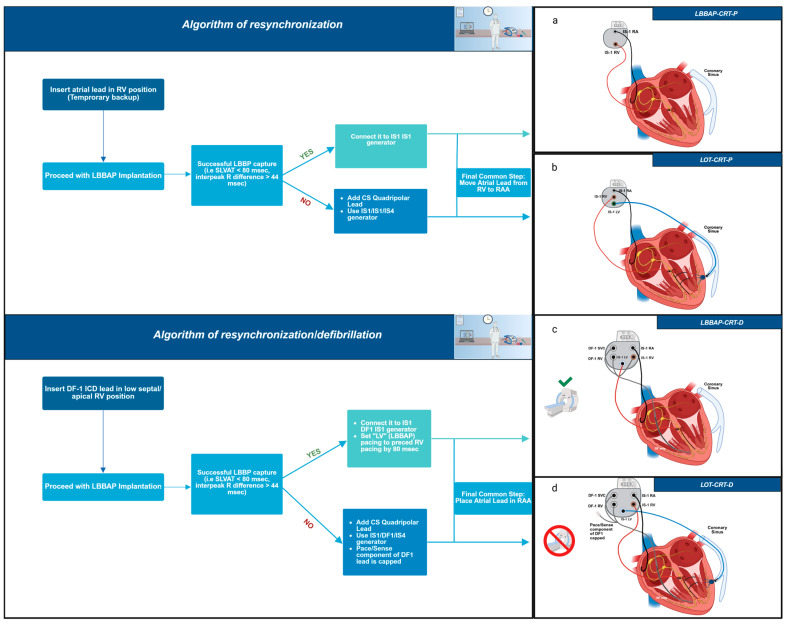
Suggested implantation algorithm for LBBAP-CRT-D and LOT-CRT-D. This stepwise depiction aids clinical decision-making depending on the resynchronization or defibrillation requirements. Lead placement is illustrated in each modality. Subfigures (**a**–**d**) depict the final lead configurations for each approach: (**a**). LBBAP-CRT-P: Left bundle branch pacing with a pacemaker device, involving a single lead positioned for conduction system pacing. (**b**). LOT-CRT-P: Left bundle branch optimized CRT approach combining LBBAP with an additional pacing lead placed in the coronary sinus to enhance ventricular synchrony. (**c**). LBBAP-CRT-D: LBBAP in conjunction with a defibrillating lead positioned in the right ventricle, used in a CRT-D system. (**d**). LOT-CRT-D: Combination of LBBAP, coronary sinus pacing, and a right ventricular defibrillating lead, forming a comprehensive CRT-D approach. Figure created in BioRender. Bozika, M. (2025) https://BioRender.com/yu83hr4 (accessed on 12 April 2025). https://app.biorender.com/.

As a final common step, the atrial lead is permanently positioned in the right atrial appendage (RAA). A complete representation of this suggested algorithm is provided in Figure 5. The LOT-ICD strategy (DF1 lead in the ICD generator with LBBAP in the RV port) is not preferred, as it renders patients MRI-incompatible due to the capped RV lead. Additionally, RV pacing in LBBAP-CRT-D is not significant, given the prolonged LV-to-RV activation delay set in the system, rendering the ICD lead functionally irrelevant in LV depolarization.

## 10. Patient’s Criteria Indicating Need for LOT-CRT

Several factors can predict the implementation of LOT-CRT, such as QRS duration, LVEDD dimension, NYHA class and the presence of a scar.

According to the study conducted by Parale et al. [33], a QRS duration > 131 ms leads to a better QRS reduction in comparison to patients with baseline QRS of <131 ms; it should be noted that QRS reduction is present in both groups but is more prominent in the group of QRS > 131 ms. In the CSPOT trial [37], a different cut-off of QRS > 171 ms was proposed, where patients showed a greater correction of the QRS with LOT-CRT in comparison to LBBAP alone.

Additionally, according to Ribes et al. [36], an LVEDD > 66 mm and NYHA Class III are strong indicators that LOT-CRT is a better treatment option. The results of the MADURAI trial demonstrated that when the myocardial scar, as quantified by MRI, exceeded 10%, the majority of patients (7 out of 11) received LOT-CRT-D. In contrast, when the scar burden was less than 10%, only 4 out of 105 patients underwent LOT-CRT [44]. This finding suggests that patients with a higher scar burden may benefit more from LOT-CRT rather than LBBAP alone.

## 11. Limitations

It is important to emphasize that the present work has been conducted as a literature review rather than a systematic review. Formal methodological frameworks such as the Preferred Reporting Items for Systematic Reviews and Meta-Analyses (PRISMA) were not followed, and we did not perform a structured study selection process. The aim of this review was to provide an overview of the existing literature, highlight key themes, and identify gaps in the current knowledge that warrant further investigation.

Additionally, the studies included in this manuscript present certain limitations. The sample size was relatively low, with limited statistical power, while selection bias was prevalent due to treatment allocation based on physicians’ discretion rather than randomization. Most of the studies were single-center, without external validation, a finding that restricts their applicability. Specific studies focused on surrogate endpoints (such as QRS duration or LVEF improvement) without providing data concerning all-cause mortality and heart failure hospitalization [31,32,33,34,35]. The CSPOT study lacked longitudinal follow-up; it included DSP patients as well as anodal capture, while patients with AV block were excluded [37]. Thus, their clinical applicability is limited. Moreover, most studies pooled the clinical outcomes of LOT-CRT patients without providing specific outcomes on LOT-CRT-D and LOT-CRT-P. Thus, there is a limited ability to draw conclusions specifically on each device and its applicability in clinical practice. Overall, these findings showcase a gap in the literature and underscore the importance of the conduction of randomized controlled trials to further evaluate the clinical significance of each modality on the population.

## 12. Conclusions

LOT-CRT is a promising modality that can be applied in clinical settings where the use of Conventional Biventricular Pacing is unsuccessful and when LBBAP alone does not deliver the expected outcomes as in atypical LBBB patterns or when LBB capture is not unsuccessful. The technique, as shown, provides enhanced QRS shortening, an increase in the percentage of the LVEF in the patients, an improvement in NYHA class, and most studies reported a reduction in NT-pro-BNP levels. The CSPOT study supports the added value of LOT-CRT, specifically in patients with complex conduction disease or suboptimal LBBAP. The provided QRS narrowing is superior without compromising the hemodynamic performance. On the other hand, when a defibrillator is needed (LOT CRTD), the device is rendered not compatible with MRI due to the capped DF 1 lead. At the same time, the procedural complexity and required expertise limit the widespread use of LOT-CRT in routine clinical practice. While it is a more sophisticated technique that demands additional time and experience, and one should consider the limitations presented by the studies, its hemodynamic and echocardiographic benefits, along with the reduction in heart failure complications, cannot be overlooked. Further data from randomized controlled trials and longer follow-up periods are necessary to better define its impact on mortality and heart failure outcomes.

## Figures and Tables

**Table 1:** A summary of the current left bundle branch block (LBBB) definitions.

Criteria	Strauss Criteria (2011)	ESC Guidelines 2021	2018 Bradycardia Clinical Practice Guidelines: Executive Summary 2018 ACC/AHA/HRS Guideline on the Evaluation and Management of Patients
QRS durations	≥130 ms for women. ≥140 ms for men.	≥120 ms	≥120 ms
QRS morphology	Broad, notched or slurred R wave. Broad, notched or slurred mid-QRS ≥ 2 of V1, V2, V5, V6, I and aVL leads.	Notches or slurring in middle third of QRS in at least two of V1, V2, V5, V6, I, and aVL. Frontal Plane: R wave in I and aVL often with negative asymmetric T wave. Horizontal plane: unique Q wave in V6 with negative asymmetrical T wave. QRS axis is variable.	Broad, notched or slurred R wave in I, avL, V1, V2, V5, V6. Occasional RS pattern in V5, V6.
Q waves	QS or rS in V1	Frontal plane: QS in aVR, with positive T wave. Horizontal plane: QS or rS in V1 with small ‘r’ with ST slightly elevated and positive asymmetrical T wave.	Absent Q wave in I, V5, V6, but in aVL a narrow Q wave may be present in the absence of myocardial pathology.
R peak time	N/A	Prolonged delayed peak in R in V5–V6 > 60 ms.	>60 ms in V5 and V6 but normal in V1, V2 and V3 with small initial R waves.
ST and T wave changes	N/A	When QRS < 140 ms, T wave in V6 may be positive. Frontal plane: ST depression. ST segment slightly opposed to QRS polarity, particularly when it is 140 ms at least and is rapidly followed by an asymmetrical T wave with also opposed polarity.	ST and T waves usually in opposite direction to QRS.

Abbreviations: ESC: European Society of Cardiology, ACC/AHA/AHRS: American College of Cardiology/American Heart Association/American Heart Rhythm Society.

**Table 2:** Summary of key clinical studies on left bundle branch-optimized therapy (LOT-CRT), including design, endpoint, outcomes.

Study Writers or Name [Year, Patients (n)]	Study Design	Study Population (Indication, N, Type of Cardiomyopathy)	Success of Procedure and LBBAP Capture	Primary and Secondary Endpoints	Outcomes
Jastrzębski et al. (2021, 112) [30]	-Prospective observational multicenter, non-randomized, single-arm -LBBAP-optimized CRT (LOT-CRT)	-Patients qualified for CRT and patients with CRT that were not responders -112 -ICM: 68/112 (61%) NICM: 44/112 (39%)	-91/112 had successful implantation -LBB capture: 68/91 (75%) LVS:23/91 (25%)	Primary endpoint: ECG, echocardiographic and clinical response at 3 months	-QRS duration: from 188 ± 26 ms to 144 ± 22 ms (*p* < 0.0001) -LVEF: from 28.5% ± 9.9% to 37.2% ± 6.12 (*p* < 0.0001). -LVEDD: from 62.0 ± 8.9 to 59.1 ± 9.1 (*p* = 0.0442) -LVEDV and LVESV: from 209.8 ± 99 to 171.4 ± 83 (*p* < 0.0001), from 149.5 ± 84 to 110.6 ± 69 (*p* < 0.0001), respectively. -NYHA: 2.9 ± 0.6 to 1.9 ± 0.6 (*p* < 0.0001) -NT-pro-BNP: from 5668 ± 8249 pg/mL to 2561 ± 3555 pg/mL (*p* < 0.0001)
Chen et al. (2022, 100) [31]	-Non-randomized, prospective observational study multicenter -LBBAP-CRT (LOT) vs. BVP-aCRT	-HFrEF with LVEF < 35% and LBBB -LBBAP-CRT: 49 BVP-aCRT: 51 -LBBAP-CRT: DCM: 36/49 (73.47%) BVP-aCRT: DCM: 41/51 (80.39%)	LBBAP-CRT: 49 -1 failed and 4 were added from BVP-aCRT	Comparison of electromechanical effects and clinical efficacy of LOT-CRT at 6 months and 1 year	-QRS duration: Fused LBBAP vs. -BVP-aCRT (102.61 ± 9.66 ms vs. 126.54 ± 11.67, *p* < 0.001) -LVEF: LBBAP vs. BiV-aCRT (47.58 ± 12.02% vs. 41.24 ± 10.56% *p* = 0.008 at 6 months and 49.10 ± 10.43% vs. 43.62 ± 11.33%, *p* = 0.021 at 1 year). In super responders LVEF had a great increase in LBBAP-CRT vs. -BVP-aCRT (from 53.06% to 61.22% vs. 36.59% to 38.22%, respectively, *p* < 0.001) -LVEDD: LBBAP vs. BiV-aCRT (57.30 ± 8.00 mm vs. 62.40 ± 10.07 ms, *p* = 0.015 at 6 months and at 1 year 54.50 ± 6.13 vs. 60.99 ± 10.68 mm, *p* = 0.001) -LVSDD: LBBAP vs. BiV-aCRT (43.40 ± 9.66 ms vs. 49.44 ± 11.51 mm, *p* = 0.014 at 6 months and at one year 41.78 ± 9.05 vs. 48.33 ± 12.63 mm, *p* = 0.007) -NYHA: LBBAP-CRT group vs. BiV-aCRT, (4.08% vs. 19.61% *p* = 0.028) at 1 year
			BVP-aCRT: 51/55 (5 were converted in LBBAP-CRT)		
Feng et al. (2022) [32]	-Prospective, observational, single center. -LOT-aCRT vs. BiV-aCRT	-HF with LVEF ≤ 35% and LBBB -LOT-aCRT: 10 BiV-aCRT: 11 -LOT-aCRT: ICM: 6/10 (60%) BiV-aCRT: ICM: 5/11 (45.5%)	LBB capture: 7/10 (70%)	Feasibility, safety and efficacy of LOT-aCRT, 9-month follow-up	-QRS duration: LOT-CRT group (unipolar LBBAP-LOT-BiV-CRT) vs. BiV-CRT group (from 168.1 ± 18.9 to 123.0 ± 5.7 ms with unipolar LBBAP, *p* = 0.01, to 121 ± 3.8 ms with LOT, *p* > 0.05 and from 158.0 ± 13.0 ms to 132.0 ± 4.5 ms *p* = 0.019 with BiV pacing vs. from 176.7 ± 19.7 ms to 133.3 ± 8.2 ms in the BiV group, *p* = 0.011) -LVEF: LOT-aCRT vs. BiV-aCRT (32.0 ± 4.2 to 45.0 ± 5.1%, *p* = 0.011 vs. 34.0 ± 1.3% to 45.8 ± 12.0% *p* = 0.143, respectively) -NT-pro-BNP: LOT-aCRT vs. BiV-aCRT (from 3240 ± 2258 pg/mL to 1151 ± 1774 pg/mL, *p* = 0.04) vs. 2684 ± 1083 pg/mL to 2066 ± 1444, *p* = 0.219 -NYHA: LOT-aCRT vs. BiV-aCRT (3.4 ± 0.55 to 2.4 ± 0.55 at 9 months, *p* = 0.032 vs. 3.3 ± 0.52 to 2.5 ± 0.55, *p* = 0.024)
Parale et al. (2023) [33]	-Prospective, Cross-sectional, single center. - LOT-CRT vs. LBBAP-CRT on the same patient population with three pacing modalities (AAI, DDD from LBBAP and DDD for LBB and LV pacing)	-NICMP with LVEF < 35% and LBBB -24 -N/A	LBB capture: 21/24 had successful LBB pacing	Primary: QRS duration Secondary: TpTe, QT interval	-QRS duration: LOT-CRT vs. LBBAP-CRT (from 167 ± 21.2 ms to 129.5 ± 18.6 ms, *p* = 0.01 vs. from 167 ± 21.2 ms to 134.5 ± 23.6 ms, *p* < 0.001, respectively) -QRS area: LOT-CRT vs. LBBAP-CRT (from 148.3 ± 80.5 μVs to 63.8 ± 34.9 μVs *p* < 0.0001 vs. from 148.3 ± 80.5 μVs to 94.2 ± 61.6 μVs, *p* < 0.001) -TpTe: LOT-CRT vs. LBBAP-CRT (from 94.6 ± 22.3 ms to 66.2 ± 21.9 ms, *p* < 0.001 vs. 94.6 ± 22.3 ms to 75.8 ± 21.2 ms, *p* < 0.001, respectively) -TpTe/QT ratio: LOT-CRT vs. LBBAP-CRT (0.22 ± 0.04 to 0.16 ± 0.04, *p* < 0.001 vs. 0.22 ± 0.04 to 0.19 ± 0.04, *p* = 0.05) -IVMD: LOT-CRT vs. LBBAP-CRT (78.3 ± 22 ms to 17.6 ± 13.8 ms, *p* < 0.001 vs. 78.3 ± 22 ms to 17.5 ± 13.9 ms, *p* < 0.001)
Chen et al. (2023, 85) [34]	-Prospective, observational. -LOT-CRT vs. BiV-CRT	-IVCD with LVEF ≤ 35% -LOT-CRT: 30 BiV-CRT: 55 -LOT-CRT: ICM: 10/30 (33.3%) BiV-CRT: ICM: 12/55 (21.8%)	LOT-CRT: 30/31 (crossover to BiV-CRT) BiV-CRT: 55/56 (one crossover from the LOT-CRT group, one failure of CS lead)	Echocardiographic and clinical characteristics at 2-year follow-up	-QRS duration: LOT-CRT vs. BiV-CRT (183.6 ± 20.3 ms to 140.9 ± 17.6 ms vs. 176 ± 19.9 ms to 154.1 ± 20.2 ms, respectively, *p* < 0.005) -LVEF: LOT-CRT vs. BiV-CRT (30 ± 7.3% to 36.7 ± 9.8% at 6 months, *p* < 0.01 and to 37 ± 9.5% at 24 months vs. 29.1 ± 6.8% to 30.5 ± 7% at 24 months) -LVESV: LOT-CRT vs. BiV-CRT (198.4 ± 74.6 mL to 161.1 ± 68.9 mL, *p* = 0.0018 vs. 203.91 ± 70.7 to 203.8 ± 68.5 mL) -LVEDV: LOT-CRT vs. BiV-CRT (275 ± 73.5 to 252.3 ± 80, *p* = 0.0494 vs. 280.4 ± 81.5 to 289.5 ± 81) -NYHA: LOT-CRT vs. BiV-CRT (2.9 to 2.2, *p* = 0.0006 vs. 2.9 to 2.6, *p* = 0.0067) -NT-pro-BNP: LOT-CRT vs. BiV-CRT (4073 to 1794 μg/mL, *p* = 0.0048 vs. 4431 to 3036, *p* = 0.0027, respectively)
Ezer et al. (2024, 68) [35]	-Prospective, observational, single center -LOT-CRT vs. BiV-CRT based on the QLV ratio	-HFrEF with IVCD (LBBB or ns-IVCD) -LOT-CRT with QLV ratio < 70%: 28 BiV-CRT with QLV ratio > 80%: 12 BiV-CRT with QLV ratio 70–80%: 28 -LOT-CRT: Ns-IVCD: 10/28 (34%) BiV-CRT: Ns-IVCD: 8/40 (20%)	LBB capture: 20/28 (71%)	ECG and echocardiographic characteristics at 12-month follow-up	-QRS duration: LOT-CRT vs. BiV-CRT (a reduction of 40.4 ± 14 ms vs. 32 ± 13 ms, *p* = 0.024, respectively) -LVEF: LOT-CRT vs. BiV-CRT (increase of 14.9 ± 8% vs. 10.3 ± 7.4% *p* = 0.001, respectively). -NYHA: LOT-CRT vs. BiV-CRT (decrease to 1.2 ± 0.5 vs. 0.8 ± 0.4, *p* = 0.031, respectively) -NT-pro-BNP: LOT-CRT vs. BiV-CRT (1863 ± 380 pg/mL vs. 1238 ± 412 pg/mL, *p* = 0.012, respectively)
			LVS: 8/28 (28%)		
Ribes et al. (2024, 38) [36]	-Prospective, observational. -LOT-CRT-P vs. LBBAP	-HF with LVEF ≤ 35% and LBBB -38 -55.3% idiopathic cardiomyopathy, 21.1% ICM	N/A	Determinations of the predictors of better QRS narrowing, 1-year follow-up	-QRS duration: LOT-CRT vs. LBBAP-CRT (from 180 ± 22 ms to 132 ± 16 ms vs. from 180 ± 22 to 152 ± 16 ms, respectively, *p* < 0.001).
CSPOT (2024, 48) [37]	-Prospective, multicenter -LOT-CRT vs. BiV-CRT vs. unipolar LBBAP/bipolar LBBAP	-IVCD and non-LBBB -48 -ICM: 14/48 (29%)	LBBAP: 27/48 (56%) DSP: 21/48 (44%)	Hemodynamic and electrocardiographic effect in CRT eligible patients	-LV dP/dt_max_: LOT-CRT increase of (25.8% [95% CI, 20.9–30.7%] and BiV-CRT 26.4% [ 95% CI, 20.2–32.6%]), unipolar LBBAP (19.3% [95% CI, 15.0–23.7%]) or bipolar LBBAP (16.4% [95% CI, 12.7–20.0%]) -QRS shortening: LOT-CRT (29.5 [95% CI, 23.4–35.6] ms) from a baseline of 171 ± 21 ms, BiV-CRT (18.5 [95% CI, 11.0–25.9] ms, *p* = 0.005) bipolar LBBAP (11.7 [95% CI, 6.4–17.0] ms, *p* < 0.001) unipolar LBBAP (11.9 [95% CI, 6.1–17.7] ms, *p* < 0.01).
BATTLE NCT 06061627 (ONGOING 2023–2027 estimated time completion)	-Prospective, multicenter, randomized controlled study -LOT-CRT vs. BiV-CRT	-HF with NICD -83 -N/A	N/A	LV systolic function in 6 months	N/A
RESCUE NCT 06148571 (ONGOING 2023–2026 estimated time completion)	-Prospective, observational, cohort study -LBBAP or LOT-CRT as a rescue therapy for BiV-CRT	-LBBB and HF -200 N/A	N/A	Acute complications at 1 year and successful LBBP	N/A

Abbreviations: LBBAP: Left Bundle Branch Area Pacing, CRT: Cardiac Resynchronization Therapy, LOT: Left Bundle Branch Optimized Therapy, ICM: Ischemic Cardiomyopathy, NICM: Non-Ischemic Cardiomyopathy, LBB: Left-Bundle-Branch, LVS: Left Ventricular Septal capture, ECG: Electrocardiographic, LVEF: Left Ventricular Ejection Fraction, LVEDD: Left Ventricular End Diastolic Diameter, LVEDV: Left Ventricle End Diastolic Volume LVSD: Left Ventricle End Systolic Volume, NYHA: New York Heart Association, NT-pro-BNP: N-terminal pro-B-type natriuretic peptide, BVP-aCRT: Biventricular Pacing with adaptive Cardiac Resynchronization Therapy, HFrEF: Heart Failure with reduced Ejection Fraction, DCM: Dilated Cardiomyopathy, HF: Heart Failure, NICMP: Non-Ischemic Cardiomyopathy, LBBB: Left Bundle Branch Pacing, TpTe: T-peak to T-end, IVMD: Intraventricular Mechanical Delay, IVCD: Intraventricular Conduction Delay, DSP: Deep Septal Pacing, CSPOT: Conduction System Pacing Optimized Treatment, LV dP/dtmax: Left Ventricle pressure maximal first derivative.

**Table 3:** Overview of procedural metrics as they were presented (fluoroscopy time, air kerma, and procedure duration) in key studies.

Study or Writer (Year)	Fluoroscopy Time (min)	Procedure’s Duration (min)
Jastrzębski et al. (2021) [30]	27.3 ± 22	N/A
Chen et al. (2022) [31]	LOT-CRT: 9.50 ± 1.99	N/A
	BiV-CRT: 13.84 ± 5.47	
Feng et al. (2022) [32]	LOT-aCRT: 29.2 ± 8.8	LOT-aCRT: 152 ± 31
	BiV-aCRT: 21.8 ± 3.1	BiV-aCRT: 122 ± 10
Parale et al. (2023) [33]	20.8 ± 6.5	121.8 ± 22.9
Chen et al. (2023) [34]	N/A	LOT-CRT: 126.5 ± 22.6
		BiV-CRT: 105.8 ± 18.1
Ezer et al. (2024) [35]	LOT-CRT: 28 ± 15	LOT-CRT: 119.5 ± 59.5
	BiV-CRT: 21 ± 8	BiV-CRT: 89 ± 56
Ribes et al. (2024) [36]	N/A	N/A
CSPOT (2024) [37]	N/A	N/A

Abbreviations: LOT-CRT: Left Bundle Branch-Optimized Cardiac Resynchronization Treatment, BiV-CRT: Biventricular Cardiac Resynchronization Treatment, N/A: Not Available. CSPOT: Conduction System Pacing Optimized Therapy.
